# Role of Regulatory B Cells in the Progression of Cervical Cancer

**DOI:** 10.1155/2019/6519427

**Published:** 2019-06-18

**Authors:** Zhifang Chen, Yuejie Zhu, Rong Du, Nannan Pang, Fengbo Zhang, Di Dong, Jianbing Ding, Yan Ding

**Affiliations:** ^1^Department of Gynecology, The First Affiliated Hospital of Xinjiang Medical University, Urumqi 830054, China; ^2^Department of Reproductive Medicine, The First Affiliated Hospital of Xinjiang Medical University, Urumqi 830054, China; ^3^Department of Hematology, The First Affiliated Hospital of Xinjiang Medical University, Urumqi 830054, China; ^4^Department of Laboratory Medicine, The First Affiliated Hospital of Xinjiang Medical University, Urumqi 830054, China; ^5^Department of Immunology, Xinjiang Medical University, Urumqi 830011, China

## Abstract

This study is to investigate the role of regulatory B (Breg) cells in cervical cancer. In total, 70 cases of cervical cancer, 52 cases of cervical intraepithelial neoplasia (CIN), and 40 normal controls were enrolled. The percentage of Breg cells was detected by flow cytometry. Serum levels of IL-10 were measured by ELISA. The correlation between Breg cells and the clinical characterizations of cervical cancer was analyzed. The inhibition effect of Breg cells on CD8^+^ T cells was tested by blocking IL-10 *in vitro*. The percentage of CD19^+^CD5^+^CD1d^+^ Breg cells and the level of IL-10 of patients with cervical cancer or CIN were significantly higher than those in the control group (*P* < 0.05). And the postoperative levels of Breg cells and IL-10 were significantly lower than the preoperative levels (*P* < 0.05). Breg cells and the IL-10 level were positively correlated in cervical cancer patients (*r* = 0.516). In addition, the Breg cell percentage was closely related to the FIGO stages, lymph node metastasis, tumor differentiation, HPV infection, and the tumor metastasis of cervical cancer (*P* < 0.05). The Breg cell percentage was negatively correlated with CD8^+^ T cells of cervical cancer patients (*r* = ‐0.669). The level of IL-10 in the culture supernatant of Bregs treated with CpG was significantly higher than that of non-Bregs (*P* < 0.05). After coculture with Bregs, the quantity of CD8^+^ T cells to secrete perforin and Granzyme B was significantly decreased, and this effect was reversed after blocking IL-10 by a specific antibody. Breg cells are elevated in cervical cancer and associated with disease progression and metastasis. Moreover, they can inhibit the cytotoxicity of CD8^+^ T cells.

## 1. Introduction

The morbidity and mortality of cervical cancer rank the second place in the female genital tract malignant tumors. Worldwide, there are nearly 50 million new cases of cervical cancer each year, and half of them died due to lack of effective treatment [1]. In recent years, the incidence of cervical cancer is high [2] and the onset age of cervical cancer becomes younger in Xinjiang, China [3]. Surgery and/or radiotherapy is the most commonly used methods for the treatment of cervical cancer [4]. Interventional chemotherapy is also an adjuvant therapy [5]. However, some cervical cancer patients cannot be completely cured with these treatment methods [6]. Recently, tumor immunotherapy has attracted much attention. However, the mechanism of immune pathogenesis in patients with cervical cancer has not been clarified, which limits the progress of immunotherapy.

The regulatory B (Breg) cell is an independent B cell subset, which plays an important role mainly in the autoimmune and inflammatory responses [7]. Besides, Breg has an immunomodulatory effect that promotes the immune escape for tumor cells [8, 9]. Studies [10, 11] have shown that Breg is closely related to the occurrence and development of tumors. In patients with liver cancer, the CD19^+^IL-10^+^ Breg level increased and was closely associated with hepatitis B e antigen and HBV-DNA copies. In addition, there are more Breg cells in patients with advanced esophageal cancer, indicating that Breg cells play an important role in the occurrence and development of esophageal cancer [12]. However, the role of Breg cells in cervical cancer has still not been determined yet.

In this study, to evaluate the role of Breg in development of cervical cancer, we analyzed the levels of Breg cells in cervical cancer patients and the relationship of Bregs with the clinical symptoms of patients. Our findings may provide a novel immunotherapeutic target for treatment of cervical cancer.

## 2. Materials and Methods

### 2.1. Healthy Volunteers and Patients

A total of 70 patients with untreated cervical cancer admitted from January 2012 to October 2016 were enrolled in this study and were assigned as the cervical cancer group. Their age was between 36 and 60 years, with an average of 45.5 ± 6.12 years old. Fifty-two patients with cervical intraepithelial neoplasia (CIN), aged from 26 to 58 years (average 44.24 ± 5.67), were also enrolled in this study as the CIN group. Forty healthy volunteers were enrolled as the control group, with an age range of 24-66 years and an average of 45.35 ± 6.17 years. Among the 70 patients with cervical cancer, 57 patients (including patients of FIGO stages I and II) underwent radical resection of cervical cancer and were followed up at 6 months after surgery. The preoperative and postoperative Breg percentages and IL-10 levels were analyzed in these patients. There was no statistically significant difference in the ages among the three groups. The diseases, including diabetes, hypertension, cardiovascular disease, pregnancy, acute/chronic infectious disease, or metastatic tumor history, were excluded from all subjects. The clinical characteristics of all subjects were shown in Tables 1 and 2. Cervical shedding cells and venous blood (5 ml) were collected from each subject. Informed consent was obtained from each patient. This study conformed to the approved institutional guidelines and was approved by the Ethical Committee of Xinjiang Medical University.

### 2.2. HPV Detection

All subjects underwent gynecological examination, and the cervical shedding cells were collected. The DNA was extracted by using the Genome Extraction Kit (Hybribio Biotech Co. Ltd., Guangdong, China). Then, the DNA was amplified by PCR amplification using a ABI7300 thermal cycler (Applied Biosystems, Foster City, CA, USA). The 21 HPV subtypes were tested with the 21 HPV GenoArray Diagnostic Kit (Hybribio, Chaozhou, China), including 13 high-risk subtypes of 16, 18, 31, 33, 35, 39, 45, 51, 52, 56, 58, 59, and 68; 5 low-risk subtypes of 6, 11, 42, 43, and 44; and 3 Chinese common subtypes of 53, 56, and CP8304.

### 2.3. Flow Cytometry Analysis and Cell Sorting

To analyze the percentage of CD4^+^ T cells and CD8^+^ T cells, the cells were simultaneously stained with 10 *μ*l of anti-CD3-FITC, anti-CD8-PE-CY7, and anti-CD4-PerCP at room temperature for 20 min. The antibodies were from BD Biosciences (Milpitas, CA, USA). After washing with PBS twice, the cells were analyzed by FACS Aria II (BD). CD8^+^ T cells were sorted from PBMCs of healthy controls by FACS Aria II (BD).

Human PBMCs were isolated from venous blood by Ficoll-Hypaque density gradient centrifugation. To analyze the percentage of Breg cells in PBMCs, antibodies of mouse anti-human CD19-PerCP, anti-CD5-FE, and anti-CDld-APC were added and incubated at room temperature for 20 min. The antibodies were from BD Biosciences. After washing with PBS twice, the cells were suspended with PBS for FACS analysis. FlowJo software was used to analyze Breg cells, which were shown as a percentage of CD19^+^CD5^+^CD1d^+^ Breg cells by gating CD19^+^ B cells.

### 2.4. Detection of Perforin and Granzyme B

The sorted Breg cells and non-Breg cells were cultured in vitro with CpG (bacteria-derived oligodeoxynucleotides; eBioscience, Waltham, MA, USA; 10 *μ*g/ml) for 48 h to stimulate the production of IL-10 [13]. Then, they were, respectively, cocultured with CD8^+^ T cells at a 1 : 3 ratio for 72 hours. The anti-IL-10 blocking antibody was added to the coculture of Breg cells and CD8^+^ T cells. For the control, CD8^+^ T cells were incubated with anti-IL-10 blocking antibody or PBS. And at the last 5 h of culture, PMA, ionomycin, and BFA were added. For intracellular staining of perforin and Granzyme B, cells were first incubated with anti-CD8-PE-CY7 monoclonal antibody at 4°C for 20 minutes and then with anti-human perforin-PE and Granzyme B-APC antibody (BD) at 4°C for 30 min in the dark. Simultaneously, the incubation with isotype antibodies (BD) was performed. After that, the cells were collected and analyzed with flow cytometry.

## 3. ELISA

The sorted Breg cells and non-Breg cells were cultured in vitro with CpG (eBioscience) to stimulate the production of IL-10 [13]. After treatment for 48 h, the culture supernatant was collected. The levels of IL-10 in serum and culture supernatant were measured according to the instructions of the ELISA kit (eBioscience, San Diego, CA, USA). The absorbance (OD) was measured at 450 nm with an ELx800 Microplate reader (BioTek, VT, USA), and IL-10 levels were calculated according to the standard curve.

### 3.1. Statistical Analysis

The data were analyzed by SPSS13.0 software (Chicago, IL, USA). All experimental data were shown as the mean ± SD. One-way ANOVA variance analysis, *t*-test, and *χ*^2^ test were used to compare the differences of the groups. The correlation analysis was performed by the Spearman correlation test. A value of *P* < 0.05 was considered to be statistically significant.

## 4. Results

### 4.1. Percentage of Breg Cells in Three Groups

**Figure 1:**
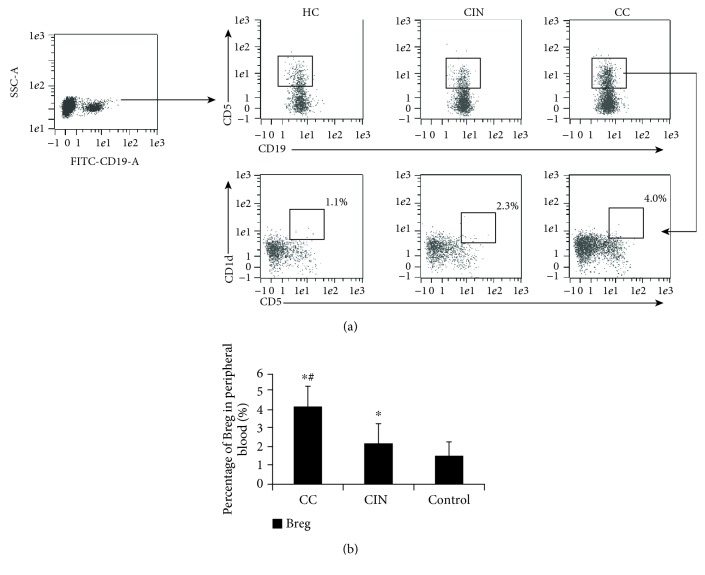
Breg cell flow cytometry. Flow cytometry was performed to detect the Breg cell percentage. (a) Representative flow cytometry results for the gating strategy of CD19^+^CD1d^+^CD5^+^ Breg cells. (b) Breg percentage in normal control, CIN, and cervical cancer groups. ^∗^*P* < 0.05 compared with the control group; ^#^*P* < 0.05 compared with the CIN group.

The percentage of CD19^+^CD5^+^CD1d^+^ Breg lymphocytes in the PBMC of healthy controls, CIN patients, and cervical cancer patients was measured by flow cytometry. The representative flow cytometry results for the gating strategy of the CD19^+^CD5^+^CD1d^+^ Breg cell population were shown in Figure 1(a). Statistically, the percentage of Breg cells in patients with cervical cancer was significantly higher than that in the CIN group and the healthy group (*P* < 0.05) (Figure 1(b)).

### 4.2. The Correlation of the IL-10 Level with the Progression of Cervical Cancer

**Figure 2:**
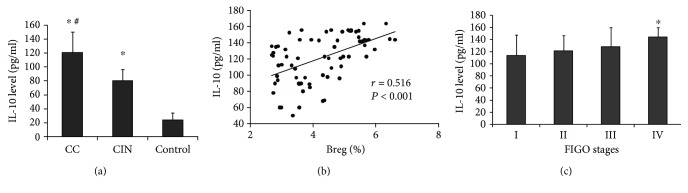
The IL-10 level in three groups and in different FIGO stages of cervical cancer patients. IL-10 level was detected with ELISA. (a) Serum IL-10 levels in three groups. ^∗^*P* < 0.05 compared with the control group; ^#^*P* < 0.05 compared with the CIN group. (b) Breg was positively correlated with IL-10 (*R* = 0.516, *P* < 0.001). (c) Serum IL-10 levels in different FIGO stages of cervical cancer patients. ^∗^*P* < 0.05 compared with stage I.

Breg lymphocytes play a negative regulatory role primarily through the secretion of IL-10. Therefore, the serum levels of IL-10 were detected by ELISA. The IL-10 level in cervical cancer patients was significantly higher than that in the CIN group and the healthy control group (*P* < 0.05) (Figure 2(a)). We further analyzed the correlation of Bregs with IL-10 in cervical cancer patients. As shown in Figure 2(b), there was a positive significant linear correlation between Bregs and IL-10 (*R* = 0.516, *P* < 0.001) (Figure 2(b)). The difference of the IL-10 level among patients with different FIGO stages (stage I (*n* = 27), stage II (*n* = 30), stage III (*n* = 8), and stage IV (*n* = 5)) was analyzed. The results showed that with the stage of cervical cancer advanced, the IL-10 level gradually increased (Figure 2(c)). Among them, the level of IL-10 in stage IV patients was significantly higher than that in stage I patients (*P* < 0.05). This result indicates that the IL-10 level in patients with cervical cancer may be closely related to the disease progression.

### 4.3. The Relationship between Bregs and Other Cell Subsets

**Figure 3:**
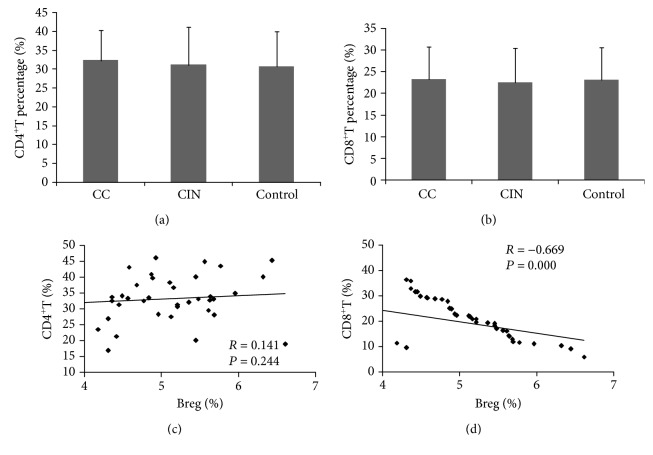
Correlation analyses between Breg and T subsets: (a) alteration of CD4^+^ T cells in patients; (b) alteration of CD8^+^ T cells in patients; (c) correlation between Breg and CD4^+^ T cells; (d) correlation between Breg and CD8^+^ T cells (*r* = ‐0.669, *P* ≤ 0.001).

In this study, we found that the percentages of CD4^+^T and CD8^+^T were not significantly different between the three groups of the cervical cancer group, the CIN group, and the control group (Figures 3(a) and 3(b)). We further analyzed the correlation of CD19^+^CD5^+^CD1d^+^ Bregs with CD4^+^ T cells and CD8^+^ T cells. Statistical analysis showed that there was no significant linear correlation between CD19^+^CD5^+^CD1d^+^ Bregs and CD4^+^ T cells (*R* = 0.141, *P* = 0.244) (Figure 3(c)). However, CD19^+^CD5^+^CD1d^+^ Bregs were negatively correlated with CD8^+^ T lymphocytes (*R* = ‐0.669, *P* ≤ 0.001) (Figure 3(d)). This result suggests that the Breg cells might regulate the function of CD8^+^ T cells in cervical cancer patients.

### 4.4. Relationship between Breg Cells and the Clinical Features of Patients with Cervical Cancer

**Figure 4:**
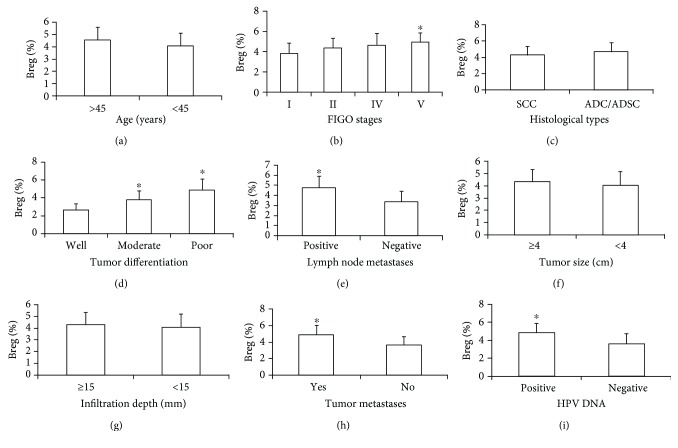
The relationship between Breg cells and clinical features of cervical cancer. The relationship between Breg cell ratios of cervical cancer patients and (a) age, (b) FIGO stages, (c) histological types, (d) tumor differentiation, (e) lymph node metastasis, (f) tumor diameter, (g) invasion depth, (h) tumor metastasis, and (i) HPV DNA was analyzed. ^∗^*P* < 0.05 means statistically significant.

We further studied the relationship of Breg cells with patient age (Figure 4(a)), FIGO stages (Figure 4(b)), histological type (Figure 4(c)), tumor differentiation (Figure 4(d)), lymph node metastasis (Figure 4(e)), tumor size (Figure 4(f)), depth of invasion (Figure 4(g)), tumor metastasis (Figure 4(h)), and HPV infection (Figure 4(i)). And we found that Bregs were closely related to FIGO stages, the lymph node metastasis, the tumor differentiation, HPV infection, and the tumor metastasis. However, there was no obvious correlation of Bregs with ages, histological type, tumor size, and depth of invasion. This result indicates that Breg cells in patients with cervical cancer may be closely related to the metastasis and degree of malignancy.

### 4.5. The Change of Bregs and IL-10 before and after Surgery

**Figure 5:**
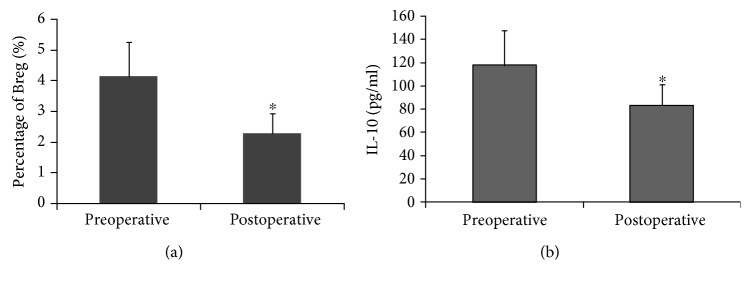
Preoperative and postoperative Breg and IL-10 in patients who underwent radical resection of cervical cancer. Among the 70 patients with cervical cancer, 57 patients underwent radical resection of cervical cancer and were followed up at 6 months after surgery. The preoperative and postoperative Breg percentages and IL-10 levels were analyzed in these patients. The number of Breg cells (a) and the level of IL-10 (b) before and after surgery were compared. ^∗^*P* < 0.05, compared with the preoperative group.

The changes of Bregs and IL-10 before and after the surgery were investigated in 57 patients who underwent radical resection of cervical cancer. Figures 5(a) and 5(b) showed that comparing to the preoperative ones, the postoperative percentage of Breg cells and the postoperative level of IL-10 were significantly decreased (4.15 ± 1.11%*vs.*2.27 ± 0.65% and 117.81 ± 29.45 pg/ml*vs.*83.28 ± 18.08 pg/ml). It indicated that Bregs and the level of IL-10 decreased in patients with surgical resection.

### 4.6. The Bregs Inhibit CD8^+^ T Cells by IL-10

**Figure 6:**
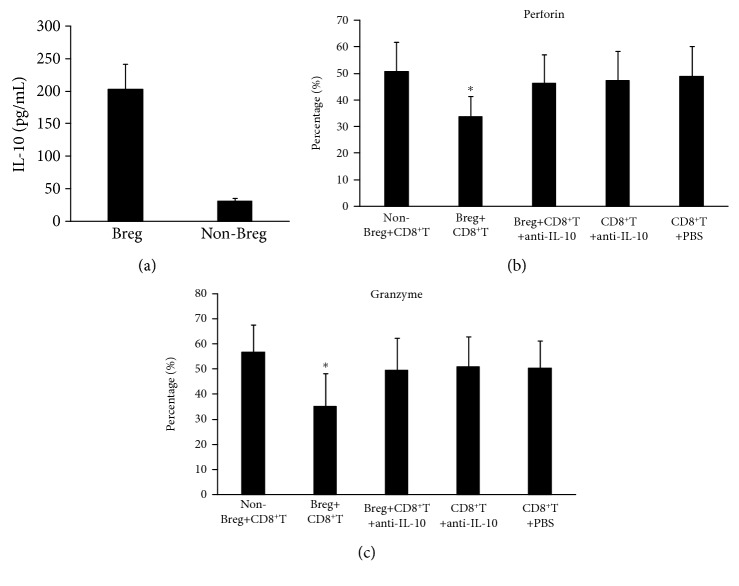
The inhibitory effect of Breg cells on CD8^+^ T cells. (a) Level of IL-10 in the culture supernatant of non-Breg and Breg cells after treatment with CpG. ^∗^*P* < 0.05 compared with the non-Breg group. (b) Perforin and (c) Granzyme level in non-Breg+CD8^+^T, Breg+CD8^+^T, Breg+CD8^+^T+anti-IL-10, CD8^+^T+anti-IL-10, and CD8^+^T+PBS groups. Breg+CD8^+^T group compared with other groups, ^∗^*P* < 0.05.

We sorted out Bregs and non-Breg with flow cytometry. The purity of Breg cells was above 95%, and the purity of non-Breg was 99.9%. To further confirm the identification of Breg, we cultured the sorted Breg cells and non-Breg cells in vitro with CpG for 48 h and then detected IL-10 production by ELISA. The result showed that Breg cells produced significantly more IL-10 (202.8 ± 39.2 pg/ml) than non-Breg cells (31.02 ± 4.58 pg/ml) (Figure 6(a)), indicating that Breg cells are successfully identified. In order to study the inhibitory effect of Bregs on CD8^+^ T cells, we cocultured two cells and detected the secretion of perforin and Granzyme B. In the presence of non-Bregs, CD8^+^ T cells were able to produce high levels of perforin and Granzyme B (50.77 ± 10.99% and 56.94 ± 10.95%, respectively) (Figures 6(b) and 6(c)). However, in the coculture of Bregs and CD8^+^ T cells, the quantity of CD8^+^ T cells to secrete perforin and Granzyme B was significantly reduced to 33.68 ± 7.63% and 35.24 ± 13.12%, respectively. And when anti-IL-10 antibody was added, the quantity of CD8^+^ T cells to secrete perforin and Granzyme B was significantly restored to 46.08 ± 10.87% and 49.64 ± 12.83%, respectively. However, anti-IL-10 antibody or PBS treatment alone did not significantly affect the secretion of perforin and Granzyme B by CD8^+^ T cells. These results suggest that Bregs may have significant inhibitory effect on the function of CD8^+^ T cells by secreting IL-10.

## 5. Discussion

The prevalence of cervical cancer in Xinjiang Uygur women (459-590/10 million) and mortality (15.78/10 million) were significantly higher than those of other ethnic groups living in the same environment [14]. Besides, the onset of cervical cancer in Xinjiang Uygur women is significantly earlier than that of other ethnic groups. Actually, the mortality of Uygur cervical cancer ranks the first among the national minorities [15].

Mizoguchi et al. found that B cells could inhibit the formation of chronic colitis, and then, the concept of Bregs was firstly proposed [16]. It has been suggested that CD1d^+^CD5^+^CD19^+^ B cells are the predominant B cell subpopulation that secretes IL-10, which is considered as one of the characteristics of Bregs [17], and thus, Bregs are also defined as B10 cells [7, 18]. In addition, Bregs may contribute to the progression of tumors [19]. However, the change and role of Bregs in cervical cancer are unknown. In order to understand the role of Bregs in patients with cervical cancer, we analyzed CD1d^+^CD5^+^CD19^+^ Breg cells and the level of IL-10. By comparing with the healthy controls and the CIN group, we found that the Breg cells and IL-10 of cervical cancer patients were significantly increased and that they were positively correlated with each other. We further studied the changes of Breg cells and the IL-10 level before and after the surgery and found that they were significantly decreased after the tumor resection. These results indicate a certain relationship of Breg cells and the IL-10 level with the occurrence and development of cervical cancer.

At the same time, we found that the percentage of Bregs and IL-10 increased with the degree of FIGO stages of cervical cancer. Kessel et al. found that Breg cells were closely related to the tumor size and portal vein invasion in the liver cancer [20]. Here, we analyzed the relationship between Breg cells and the clinical features of patients with cervical cancer. It was found that Bregs were not only related to the FIGO stages but also closely associated with lymph node metastasis, tumor differentiation, HPV infection, and the tumor metastasis in cervical cancer patients. Therefore, the increase of Bregs may be closely related to a poor outcome of the cervical cancer. Bregs may be used as an indicator for evaluating the development of cervical cancer.

The host CD4^+^ T cells and CD8^+^ T cells play an important role in the occurrence, development, and metastasis of tumors. Here, we found that Breg cells were negatively correlated with CD8^+^ T cells, which suggest that Breg cells may be involved in the immunoregulation and inhibition of CD8^+^ T cells in the cervical cancer immune responses. Study [21] demonstrates that Breg promotes tumorigenesis by inhibiting T cell killing during tumor metastasis. To confirm the role of Breg in CD8^+^ T cells, Breg cells and non-Breg cells were sorted out. The sorted Breg cells and non-Breg cells were further verified by detecting IL-10 production. We found that the levels of IL-10 in culture supernatant of Bregs treated with CpG were higher than those of non-Bregs, indicating that Breg cells and non-Breg cells are successfully identified and that Breg cells in cervical cancer patients may exert their regulatory effects through IL-10. Then, Breg cells and non-Breg cells were cocultured with CD8^+^ T cells. The results found that the quantity of CD8^+^ T cells to secrete perforin and Granzyme B was significantly decreased when CD8^+^ T cells were cocultured with Breg cells. In the host immune responses against pathogen infection and tumor, CD8^+^ T cells exert cytotoxicity against target cells by releasing perforin and Granzyme B [19, 22, 23]. Study has found that Breg cells played an immunosuppressive role in the pathogenesis of infection and could inhibit the effect of CD8^+^ T cells [24]. Breg cells also could inhibit liver inflammation by secreting IL-10 and could abrogate the viral-specific CD8^+^ T cell responses [25]. Inoue et al. [26] found that IL-10 produced by B lymphocytes inhibited the ability of CD8^+^ T cells to kill tumor cells. Next, to determine the effect of IL-10 secreted from Breg on CD8^+^ T cells, we cocultured CD8^+^ T cells and Breg cells with anti-IL-10. The results showed that the quantity of CD8^+^ T cells to secrete perforin and Granzyme B was significantly restored. These results suggest that Bregs may play an immunosuppressive role in the progression of cervical cancer.

## 6. Conclusions

Our study found that Breg cells in cervical cancer patients were significantly increased, which was closely related to disease progression. Breg cell-mediated inhibition on immune activation CD8^+^ T cells is thought to contribute to the development of cervical cancer. We propose that reducing Breg or blocking its function in patients with cervical cancer may be used as a new treatment for antitumor immune therapy.

## Figures and Tables

**Table 1:** The clinical information of the study cohort.

	Normal control group (*n* = 40)	CIN group (*n* = 52)	Cervical cancer group (*n* = 70)
Age	24-66	26-58	36-60
Ethnic group	Uighur	Uighur	Uighur
HPV	8	24	59 (84.2)
	32	28	11 (15.8)

**Table 2:** The clinical characteristics of the cervical cancer patients.

Items	Category	*N* = 70
Age	<45	14 (20)
	>45	56 (80)

FIGO stage	I	27 (38.5)
	II	30 (42.8)
	III	8 (11.4)
	IV	5 (7.14)

Histology type	SCC	60 (85.7)
	ADC/ADSC	10 (14.3)

Tumor differentiation	Well	9 (12.8)
	Moderate	25 (35.7)
	Poor	36 (51.4)

Lymph node metastases	Positive	23 (32.8)
	Negative	47 (67.2)

Tumor size (cm)	<4	39 (55.7)
	≥4	31 (44.3)

Infiltration depth (mm)	<15	37 (52.8)
	≥15	33 (47.2)

Tumor metastasis	Yes	24 (34.2)
	No	43 (61.4)
	Unknown	3 (4.4)

HPV DNA	Positive	59 (84.2)
	Negative	11 (15.8)

Surgical treatment	Yes	57 (81.4)
	No	13 (18.6)

## Data Availability

The data used to support the findings of this study are available from the corresponding author upon request.
